# Inspection of Ureteral Orifices: The Pearl of Flexible Cystoscopy

**DOI:** 10.1089/cren.2015.29011.smi

**Published:** 2015-10-01

**Authors:** Seshikanth Middela, Charmaine Matthews, Hamid Bushra, Sanjay Das, Bo Pettersson

**Affiliations:** Countess of Chester Hospital NHS Foundation Trust, Chester, United Kingdom.

## Abstract

Cystoscopy is most common diagnostic investigation. The examination technique and the findings, both normal and pathological, were well described described a hundred years ago. With technological advances, there has been over-emphasis on imaging modalities for diagnostic purposes. A basic maneuver of examining the ureteral orifices is sometimes rushed through when in fact careful examination can clinch the diagnosis. The importance is exemplified by two cases, one of which is a rare case of Xanthoma of the ureter.

## Introduction

Cystoscopy is the most commonly used diagnostic investigation in urology. The humble cystoscope has come a long way since its inception in 1805. On the way it has undergone various anatomic modifications and physiologic and technologic advances to the present-day flexible cystoscope. The instrument may have undergone transformation, but in this modern age of positron emission tomography (PET) scanners and narrow band imaging (NBI) the basic technique of bladder inspection still remains the same as described in the literature a hundred years ago. One of the important steps emphasized in the literature is the visual inspection of the ureteral orifices with regard to the appearance, peristalsis, and efflux of urine. We present two interesting cases emphasizing the careful inspection of the ureteral orifices.

## Case Number 1

**FIG. 1.**
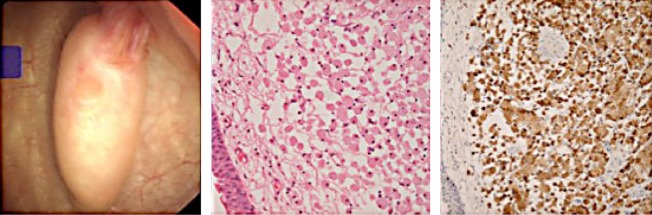
Xanthoma HPE CD68 stain.

A 38-year-old male was referred for frank hematuria. Flexible cystoscopy showed a normal bladder mucosa, but careful inspection of the left ureteral orifice revealed a pedunculated growth with smooth mucosal surface prolapsing with every peristalsis (Fig. 1). He underwent ureteroscopic resection of the same, which microscopically turned out to be a xanthoma of the ureter. This was not picked up on the CT urogram. This is the first known immune-stained proven case report of a ureteral xanthoma reported in the literature. He remains recurrence free 3 years after regular ureteroscopic examinations.

## Case Number 2

**FIG. 2.**
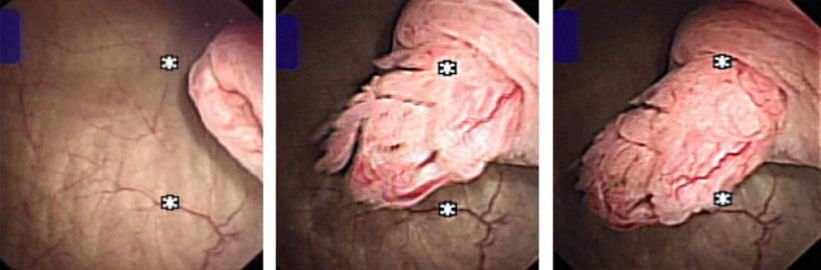
The lengthening of the distal ureteral tumor with every peristalsis.

A 65-year-old female smoker was seen by us in the one-stop hematuria clinic. The initial cystoscopic examination was negative, but scrutiny for the left ureteral efflux revealed a gradual protrusion of a polypoidal lesion with abnormal frond-like appearance. It only lengthened with every peristalsis and gradually retracted into the ureteral orifice (Fig. 2). Again this was not clearly seen on contrast radiology studies. The patient underwent resection of distal ureter and re-implantation of the ureter. The resected lesion was found to be a superficial transitional-cell carcinoma. She remains recurrence free to date.

## Discussion

**FIG. 3.**
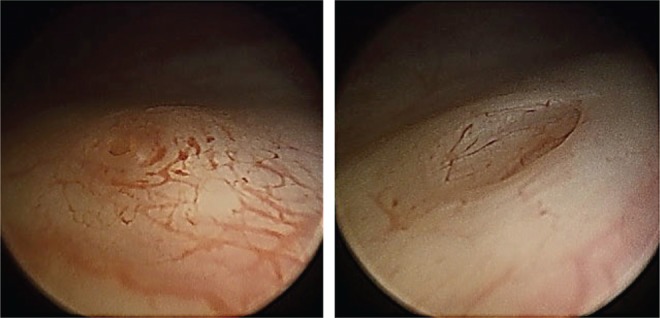
Different looking right and left ureteral orifices in the same individual. CT urogram and retrograde studies have proven normal orifices.

**FIG. 4.**
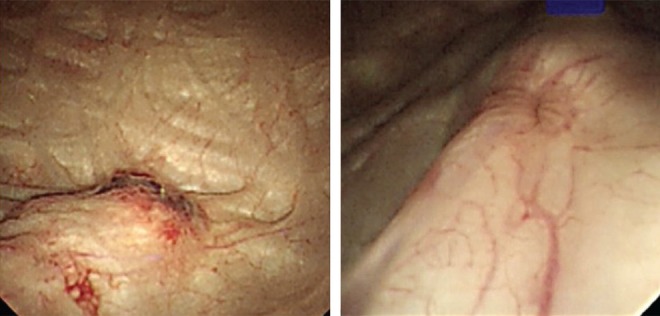
The left ureteral orifice before (inflamed and raised) and after passage of the stone.

**FIG. 5.**
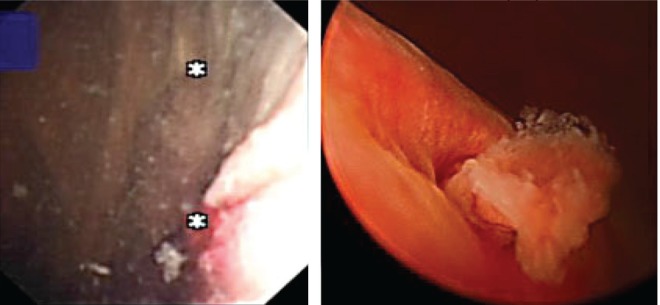
Blood ooze from right ureteral orifice tiny frond—A low-grade TCC of the renal pelvis. TCC, transitional-cell carcinoma.

Cystoscopy is an essential investigation in the diagnosis of bladder and ureteric pathology and can complement radiology. The days of rectangular prism, Bunsen's battery, and the rigid cystoscope to examine a bladder a century ago have long been gone and have given way to the modern deflectable flexible endoscope. In this age of robotic surgery and NBI and PET scanners, the art of cystoscopy remains the same and a closer inspection of the ureteral orifices is sometimes rushed through. The above described cases are a few examples of many demonstrating the value of this part of the examination. Mr. Willy Meyer, in 1888, wonderfully described the ureteral orifices, peristalsis, and the efflux before the Section of Surgery of the New York Academy of Medicine.^1^ It was predicted that with a cystoscope, the side of the diseased kidney could be proven. A definite scheme of bladder inspection is followed so as not to miss any part of the bladder. Following the pattern described,^1,2^ the ureteral orifices are inspected noting the position, shape, size, surrounding mucosa, nature, and frequency of the efflux. There may be some anatomic variation (Fig. 3), but careful inspection may also give some clues (Figs. 4 and 5).

The extremely distal ureteral lesions tend to take the shape of the ureter and can extrude and retract with every peristalsis, as described in the story of Albona Jaybis.^3^ The Xanthoma described above showed staining with CD68 and was negative with S100 indicating Xanthomatous in origin. This is the only case of Xanthoma arising from the Ureter proven on immunostaining. The orifices are easily examined by the modern flexible cystoscope; the only difficulty would be when there is a large intravesical protrusion of the median lobe when the tip cannot be flexed. Even in these scenarios, the J-maneuver can be employed for visualization of the orifices. With the J-maneuver, the bladder is filled to near capacity and the scope pushed slightly further in and flexed to 180 degrees so that it looks upon itself. It is a way of looking at the bladder neck. Modern deflectable flexible cystoscopy certainly helps in easier inspection and also allows some therapeutic interventions,^4^ but the basic principles remain the same. Judicious inspection of the ureteral orifices will accomplish the completeness of the examination and may help to pick up distal ureteral lesions at an earlier stage.

## Abbreviations Used

CT: computed tomography
NBI: narrow band imaging
PET: positron emission tomography
TTC: transitional-cell carcinoma
